# Enhanced Oxygen Utilization Efficiency With Concomitant Activation of AMPK-TBC1D1 Signaling Nexus in Cyclophilin-D Conditional Knockout Mice

**DOI:** 10.3389/fphys.2021.756659

**Published:** 2021-12-08

**Authors:** Jeejabai Radhakrishnan, Alvin Baetiong, Raúl J. Gazmuri

**Affiliations:** ^1^Resuscitation Institute, Rosalind Franklin University of Medicine and Science, North Chicago, IL, United States; ^2^Department of Clinical Sciences, Rosalind Franklin University of Medicine and Science, North Chicago, IL, United States; ^3^Captain James A. Lovell Federal Health Care Center, North Chicago, IL, United States

**Keywords:** oxygen consumption, conditional knockout, cyclophilin-D, exercise capacity, treadmill exercise

## Abstract

We have previously reported in HEK 293 T cells and in constitutive cyclophilin-D (Cyp-D) knockout (KO) mice that Cyp-D ablation downregulates oxygen consumption (VO_2_) and triggers an adaptive response that manifest in higher exercise endurance with less VO_2_. This adaptive response involves a metabolic switch toward preferential utilization of glucose *via* AMPK-TBC1D1 signaling nexus. We now investigated whether a similar response could be triggered in mice after acute ablation of Cyp-D using tamoxifen-induced ROSA26-Cre-mediated (i.e., conditional KO, CKO) by subjecting them to treadmill exercise involving five running sessions. At their first treadmill running session, CKO mice and controls had comparable VO_2_ (208.4 ± 17.9 vs. 209.1 ± 16.8 ml/kg min^−1^), VCO_2_ (183.6 ± 17.2 vs. 184.8 ± 16.9 ml/kg min^−1^), and RER (0.88 ± 0.043 vs. 0.88 ± 0.042). With subsequent sessions, CKO mice displayed more prominent reduction in VO_2_ (genotype & session interaction *p* = 0.000) with less prominent reduction in VCO_2_ resulting in significantly increased RER (genotype and session interaction *p* = 0.013). The increase in RER was consistent with preferential utilization of glucose as respiratory substrate (4.6 ± 0.8 vs. 4.0 ± 0.9 mg/min, *p* = 0.003). CKO mice also performed a significantly higher treadmill work for given VO_2_ expressed as a power/VO_2_ ratio (7.4 ± 0.2 × 10^−3^ vs. 6.7 ± 0.2 10^−3^ ratio, *p* = 0.025). Analysis of CKO skeletal muscle tissue after completion of five treadmill running sessions showed enhanced AMPK activation (0.669 ± 0.06 vs. 0.409 ± 0.11 pAMPK/β-tubulin ratio, *p* = 0.005) and TBC1D1 inactivation (0.877 ± 0.16 vs. 0.565 ± 0.09 pTBC1D1/β-tubulin ratio, *p* < 0.05) accompanied by increased glucose transporter-4 levels consistent with activation of the AMPK-TBC1D1 signaling nexus enabling increased glucose utilization. Taken together, our study demonstrates that like constitutive Cyp-D ablation, acute Cyp-D ablation also induces a state of increased O_2_ utilization efficiency, paving the way for exploring the use of pharmacological approach to elicit the same response, which could be beneficial under O_2_ limiting conditions.

## Introduction

Cyclophilin-D (Cyp-D) is a mitochondrial matrix-resident peptidyl-prolyl isomerase involved in various pleotropic functions of the cell including energy metabolism, metabolic adaptation, and nuclear/mitochondria signaling, and recently in nuclear translocation of apoptosis inducing factor (Elrod et al., 2010; Tavecchio et al., 2013; Tubbs et al., 2014; Radhakrishnan et al., 2015, 2019; Qamar et al., 2021). We first reported in HEK 293 T cells that Cyp-D interacts with mitochondrial transcription factors and that Cyp-D silencing downregulates the expression of mitochondrial genes initiated from the heavy strand promoter 2 (HSP2) encoding respiratory complex proteins leading to a reduction in oxygen consumption (VO_2_; Radhakrishnan et al., 2015). Further work in our laboratory showed that Cyp-D ablation using constitutive Cyp-D knockout (KO) mice also downregulated respiratory complex I and IV activities and thereby VO_2_ (Radhakrishnan et al., 2015). The VO_2_ downregulation was accompanied by a reduction in VCO_2_ but to a lesser extent resulting in an increased respiratory exchange ratio (RER) consistent with a metabolic shift favoring glucose utilization over fat. This effect was accompanied by enhanced exercise capacity demonstrating increased oxygen utilization efficiency (Radhakrishnan et al., 2019). The response was mediated in part *via* adenosine monophosphate-activated protein kinase (AMPK) and its downstream partner tre-2/USP6, BUB2, cdc16 domain family member 1 (TBC1D1), the so-called “AMPK-TBC1D1 signaling nexus.”

We now investigated whether a similar adaptive response could be triggered in animals developed with normal Cyp-D expression after acutely inducing the Cyp-D ablation. To address this question, we used a conditional KO (CKO) mouse – tamoxifen-induced ROSA26-Cre-mediated – and asked whether the acute Cyp-D ablation in CKO mice also: (i) reduces VO_2_ and improves exercise capacity, (ii) elicits a metabolic shift favoring glucose utilization, and (iii) whether AMPK-TBC1D1 signaling is involved. If the metabolic shift could be induced in adult mice born without the defect, the possibility of pharmacological manipulation became attractive, specially using small molecules to selectively destabilize the interaction between Cyp-D and mitochondrial transcription factors without necessarily affecting other Cyp-D functions and be beneficial for oxygen limiting clinical conditions.

## Materials and Methods

The studies were approved by the Institutional Animal Care and Use Committee at Rosalind Franklin University of Medicine and Science (Protocol B17-20) and by the Edward Hines VA Hospital Institutional Animal Care and Use Committee (Protocol H17-014).

### Animals

Mice were purchased from the Jackson Laboratory (Bar Harbor, ME, United States) and conditional Cyp-D KO mice were generated as described below in the Methods section. Mice were bred and group-housed in the Biologic Resource Facility (accredited by the Association for Assessment and Accreditation of Laboratory Animal Care) at the Rosalind Franklin University of Medicine and Science. Lights were set at the recommended illumination levels with a 12/12-h cycle controlled *via* automatic timers and the temperature maintained between 68 and 74°F. Mice were fed high-quality commercial laboratory diets *ad libitum*.

### Materials

NativePAGE Novex Bis-Tris gels (3–12%), NativePAGE running buffer, NuPAGE Bis-Tris gels (4–12%), NuPAGE MOPS SDS running buffer, NuPAGE transfer buffer, PVDF membrane, and SuperSignal West Femto maximum sensitivity substrate were obtained from Thermo Fisher Scientific Inc. (Brookfield, WI, United States). The antibodies phospho-AMPK (Thr 172), phospho-TBC1D1 (Ser 660), and β-tubulin were from Cell Signaling Technology (Danvers, MA, USA). Antibody for peroxisome proliferator activator receptor-γ coactivator 1α (PGC-1α) was from Santa Cruz Biotechnology (Santa Cruz, CA, United States). All other fine chemicals were obtained from Sigma-Aldrich (St. Louis, MO, United States).

### Study Design

Experiments were conducted in intact animals to assess the effects of conditional Cyp-D ablation *in vivo* on oxygen metabolism by measuring VO_2_ and derived parameters at rest and during exercise. Two groups of mice, age and gender matched, representing control (*n* = 10) and conditional Cyp-D KO (*n* = 10), were subjected to a treadmill running protocol until exhaustion, during which VO_2_, carbon dioxide production (VCO_2_), and RER, total running time before exhaustion, total running distance, and work performed were measured. Fuel utilization was calculated using VO_2_ and VCO_2_ data. Five running sessions were performed. After the running sessions, mice skeletal muscle tissues were harvested, quickly frozen in liquid N_2_, and stored at −80°C for Western blotting.

## Methods and Measurements

### Generation of Conditional Ppif Knockout Mice

The Jackson Lab has cryopreserved sperm (stock 005737, Ppiftm1Mmos/J) that was heterozygous for the Cyp-D floxed allele (Ppif^Fl/+^). This stock was originally generated by Dr. Stanley J. Korsmeyer’s lab (Schinzel et al., 2005). Jackson Lab performed a cryorecovery from the frozen sperm using wild-type female (stock 000664, C57BL/6J) as oocyte donors. A heterogeneous Ppif colony (Ppif^Fl/+^) was generated by the Jackson Lab.

Ppif^Fl/+^ mice from two different donating mothers were inter-crossed to generate homozygous Cyp-D floxed mice (Ppif^Fl/Fl^). Then, Ppif^Fl/Fl^ mice were crossed with mice homozygous for the ROSA26-Cre^ERT2^ (Cre/Cre) transgene (stock 008463; Gt(ROSA)26Sortm1(cre/ERT2)Tyj) to generate mice heterozygous for the Cyp-D floxed allele and hemizygous for ROSA26-Cre^ERT2^ transgene (Ppif^Fl/+^-Cre/^+^). Next, the Ppif^Fl/+^-Cre/^+^ mice were crossed with Ppif^Fl/Fl^ mice to generate conditional Cyp-D knockout mice (Ppif^Fl/Fl^-Cre/^+^) and controls (Ppif^Fl/Fl^).

### Genotyping

Genotyping was performed according to the recommendations by the Jackson Lab. Briefly, tail biopsy (~2 mm) from 21-day-old pups were digested overnight at 55°C using 0.5 ml/sample of DNA isolation buffer (100 mM Tris.Cl, pH 8, 5 mM EDTA, 0.2% SDS, and 200 mM NaCl) containing 5 μl of 20 mg/ml proteinase K. The samples were then heated at 95°C for 10 min to inactivate Proteinase K. Equal volume of phenol: CHCl_3_: IAA mixture was then added, mixed gently for 5 min to allow precipitation of proteins, and centrifuged at 12, 000 *g* for 5 min at 4°C for phase separation. The supernatant was carefully removed, 2 volumes of 100% ethanol were added, incubated at room temperature for 5 min, and then centrifuged at 12,000 *g* for 5 min at 4°C for DNA precipitation. The supernatant was discarded, and the pellet (DNA) was washed with 1 ml of 70% ethanol. The pellet was then dried at 37°C for 5 min and dissolved in TE buffer. The DNA concentration was determined by absorbance at 260 nm and 150 ng of DNA was used for the PCR. Two PCR reactions per sample were performed: one to detect Cre gene product and another one to detect Flox gene product. For Cre PCR, the primers used were as: oIMR 3621 (F: 5' to 3' CGT GAT CTG CAA CTC CAG TC) and oIMR 9074 (R: 5' to 3' AGG CAA ATT TTG GTG TAC GG). For Flox PCR, the primers used were as: oIMR 5116 (F: 5' to 3' GCT TTG TTA TCC CAG CTG GCG C) and oIMR 5115 (R: 5' to 3' TTC TCA CCA GTG CAT AGG GCT CTG). PCR was performed in 12 μl of reaction volume using the kit (KAPA2G Robust Hotstart Readymix PCR kit) using an applied biosystems thermal cycler (GeneAmp PCR system 2700). The thermal cycler conditions were set according to the genotyping protocol for mice stock # 005740 by the Jackson Lab and the PCR products separated using 0.7% agarose gel electrophoresis.

### Tamoxifen Injection

Tamoxifen base (T5648, Sigma) was dissolved in peanut oil (10 mg/ml) and was given as 40 mg/kg as described by Andersson et al. (2010). Eight-week-old Ppif^Fl/Fl^-Cre^/+^ mice (Flox/flox/cre, Cyp-D CKO) and Ppif^Fl/Fl^-Cre^/−^ mice (Flox/flox, control mice) received intraperitoneally 1 mg of tamoxifen per day for five consecutive days. Administration of tamoxifen induces activation of the ubiquitously expressed Cre^ERT2^ recombinase, which stimulates recombination and excision of floxed Cyp-D in all tissues. Since Ppif^Fl/Fl^ mice do not contain Cre gene, they will not undergo Cyp-D recombination and serve as control.

### Treadmill Running

**Figure 1:**
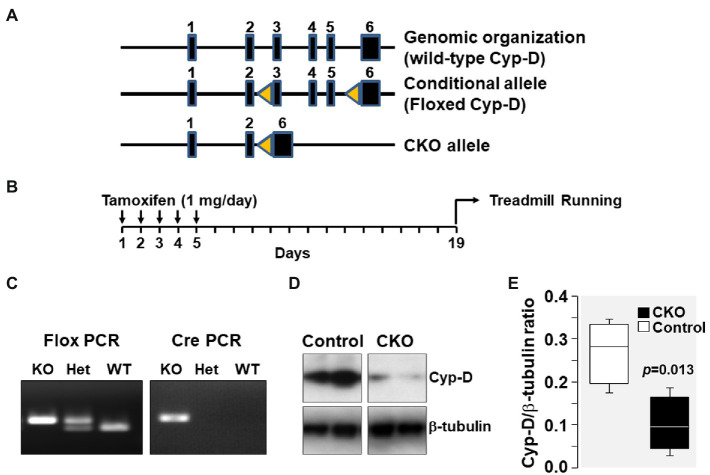
**(A)** Genomic organization of Cyp-D encoding ppif gene in mouse chromosome 14 is shown. The ppif gene has six exons which are represented by closed boxes. Genomic organization of floxed Cyp-D in which exons 3–5 has flanking lox-P sites (yellow triangles) is shown as conditional allele. The floxed Cyp-D mouse was generated by Stanley J. Korsmeyer’s lab. Genomic organization of conditional knockout (CKO) allele showing deletion of exons 3–5. **(B)** Schematic demonstrating time of tamoxifen treatment. **(C)** Ppif^Fl/+^-Cre^/+^ mice pups were genotyped to confirm the presence of Ppif^Fl/+^ and Cre genes. **(D)** Western blot showing Cyp-D protein in skeletal muscle tissue homogenates of control and cyclophilin-D conditional knockout (CKO) mice. **(E)** Box plots denote densitometry of Cyp-D normalized to β-tubulin.

After 2 weeks of completion of tamoxifen injections (i.e., on day 19 as shown in Figure 1), treadmill running was started to assess effects on oxygen metabolism (i.e., VO_2_, VCO_2_, and RER) in CKO and control mice at rest and during treadmill exercise using a calorimeter (Oxymax System, Columbus Instruments).

Treadmill running had two components: (1) Training protocol and (2) Treadmill exercise protocol.

#### Treadmill Training Protocol

It was performed as previously described (Radhakrishnan et al., 2019). Each mouse was first subjected to a 3-day habituation period. On day 1, the mouse ran 10 min at 10 m/min without inclination; on day 2, the mouse ran 10 min at 10 m/min with 20 degree inclination; and on day 3, the mouse ran 20 min at the same speed and inclination. An electrical grid placed at the start of the treadmill ramp delivered an aversive shock when the mouse stopped running and slid into the grid to elicit avoidance of subsequent shocks by running (i.e., avoidance conditioning). The mouse was discarded from the study if more than three shocks in one session were required to promote running.

#### Treadmill Exercise Protocol

It was performed as previously described (Pederson et al., 2005; Radhakrishnan et al., 2019). Each mouse ran for a total of five sessions with the treadmill at a constant 20° inclination. At the start of each session, the mouse was allowed to acclimate in the chamber for 30 min before running. Baseline measurements were obtained between min 25 and 30. The treadmill was then started at 10 m/min and its speed increased by 2 m/min every 4 min to a maximum of 26 m/min for session one and to 30 m/min in sessions two through five. The mouse was allowed to run at the maximum speed for up to 90 min or until exhaustion. The strength of the shock was kept at 2 mA for the first 30 min running at maximum speed, after which the current was reduced to 0.5 mA. Exhaustion was defined as sliding into the shock grid and sustaining a shock (0.5 or 2 mA) for a minimum of 2 s for the third time or sustaining a single shock of ≥5 s (instead of running). The investigators were blind to the mice genotype. The treadmill was cleaned between animals with 70% ethanol, wiped with napkins, and air dried. Each running session was completed by the mice cohorts (20 mice total) in 4 days. There was a 3-day resting period afterward resulting in a week for completion of one running session by the mice cohorts. It took a total of 32 days to complete the five sessions.

#### VO_2_, VCO_2_, and Derived Measurements

The measurements were performed as previously described (Radhakrishnan et al., 2019). Power (Joules/min) was calculated by dividing work performed (Joules) by time spent on treadmill (min). Fuel utilization (carbohydrate, g/min and fat, g/min) was calculated using VO_2_ and VCO_2_ data as previously described (Radhakrishnan et al., 2019) according to Carbohydrate (mg/min) = [−3.226 g carbohydrate/LO_2_ × VO_2_ (L/min) + 4.585 g carbohydrate/LCO_2_ × VCO_2_ (L/min)] × 1,000; Fat (mg/min) = [1.695 g fat/LO_2_ × VO_2_ (L/min) − 1.701 g fat/LCO_2_ × VCO_2_ (L/min)] × 1,000 (Petrosino et al., 2016).

#### Western Blotting

The Western blotting was performed as previously described (Radhakrishnan et al., 2019).

### Statistical Analysis

Linear mixed effect model was used to assess the effect of genotype and running sessions and their interaction on metabolic variables treating running sessions as discrete variable (SPSS 24.0, IBM Corp.; Armonk, NY, United States). Student’s *t*-test was used to assess (i) the effect of genotype on metabolic variables collected at baseline and at exercise and (ii) the effect of genotype on molecular signaling implementing t-test (when normality test passed) and Mann-Whitney Rank Sum test (when normality test failed; SigmaPlot v.12.5). Strength of relationships between variables was assessed by linear regression and Pearson’s product moment correlation analysis (SigmaPlot v.12.5). Data are presented as means ± SD in the text and means ± SEM in the figures. A two-tail value of *p* ≤ 0.05 was considered significant.

## Results

### Confirmation of Cyp-D Knockout

Genomic organization of the mouse genome at chromosome 14 and the strategy for conditional KO generation is shown in Figure 1. As mentioned, Ppif^Fl/+^-Cre^/+^ mice have Ppif allele flanked between two lox-P sites which upon tamoxifen injection will undergo Cre-mediated recombination resulting in deletion of the Ppif allele. Ppif^Fl/+^-Cre^/+^ mice pups were genotyped to confirm the presence of Ppif^Fl/+^ and Cre genes. Results showed that the products of expected size were generated by flox PCR (400 bp) and by Cre PCR (200 bp). Tamoxifen injection resulted in Cre-mediated deletion of lox-P flanked Ppif allele and was confirmed by Western blotting detection of Cyp-D levels in mice skeletal muscle tissue homogenates (Figure 1).

### Effect of Conditional Cyp-D Knockout on VO_2_, VCO_2_, and RER

**Figure 2:**
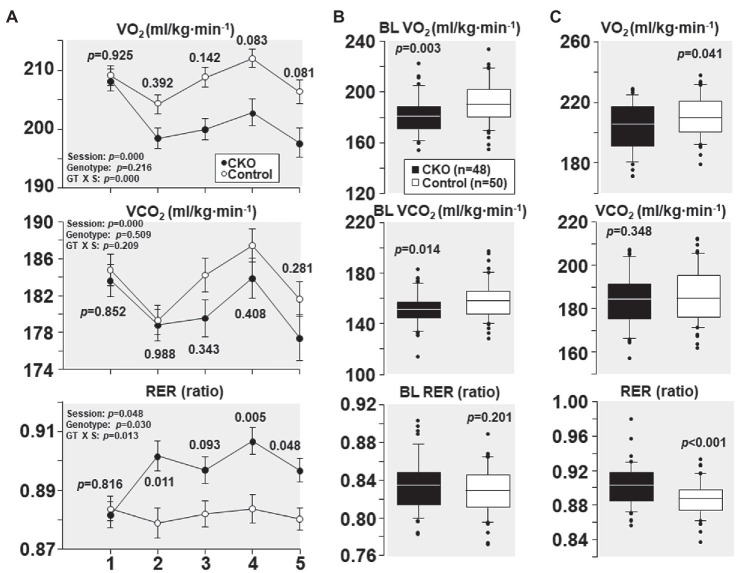
**(A)** Oxygen consumption (VO_2_), carbon dioxide production (VCO_2_), and respiratory exchange ratio (RER) of cyclophilin-D conditional knockout (CKO; *n* = 10) and control mice (*n* = 10) at five running sessions. Values are shown as mean ± SEM. Differences were analyzed by a linear mixed-effects model. Data from one CKO mouse were lost at running sessions four and five due to refusal of running. **(B)** All individual oxygen consumption (VO_2_), carbon dioxide production (VCO_2_), and respiratory exchange ratio (RER) measurements obtained under baseline (BL) conditions in cyclophilin-D conditional knockout (CKO) and control mice are shown as box plots, comparing the groups using t-test. **(C)** Box plots depicting all individual data points of oxygen consumption (VO_2_), carbon dioxide production (VCO_2_), and respiratory exchange ratio (RER) during exercise at five running sessions by conditional cyclophilin-D knockout (CKO) and control mice. Differences were analyzed by t-test.

At their first treadmill running session, as shown in Figure 2A, CKO mice and controls had comparable VO_2_, VCO_2_, and RER. However, with subsequent sessions, the VO_2_ decreased in CKO mice without a significant change in VCO_2._ The lower VO_2_ for a given VCO_2_ manifested in a significantly higher RER that remained statistically significant for most of the sessions (Figure 2A). This behavior is consistent with a metabolic adaptation induced by exercise training in CKO mice.

All individual VO_2_, VCO_2_, and RER measurements obtained at baseline – before starting treadmill exercise – combining the five sessions are shown in Figure 2B. CKO mice had a lower VO_2_ (*p* = 0.003) and a lower VCO_2_ (*p* = 0.014) but of lesser magnitude yielding a slightly higher RER which did not attain statistical significance (*p* = 0.201).

All individual VO_2_, VCO_2_, and RER measurements obtained during treadmill running combining the five sessions are shown in Figure 2C. CKO mice also exhibited a lower VO_2_ (*p* = 0.04) but without a significant difference in VCO_2_ (*p* = 0.348), yet with a highly significant increase in RER (*p* < 0.001).

### Effect of Conditional Cyp-D Knockout on Exercise Capacity

**Figure 3:**
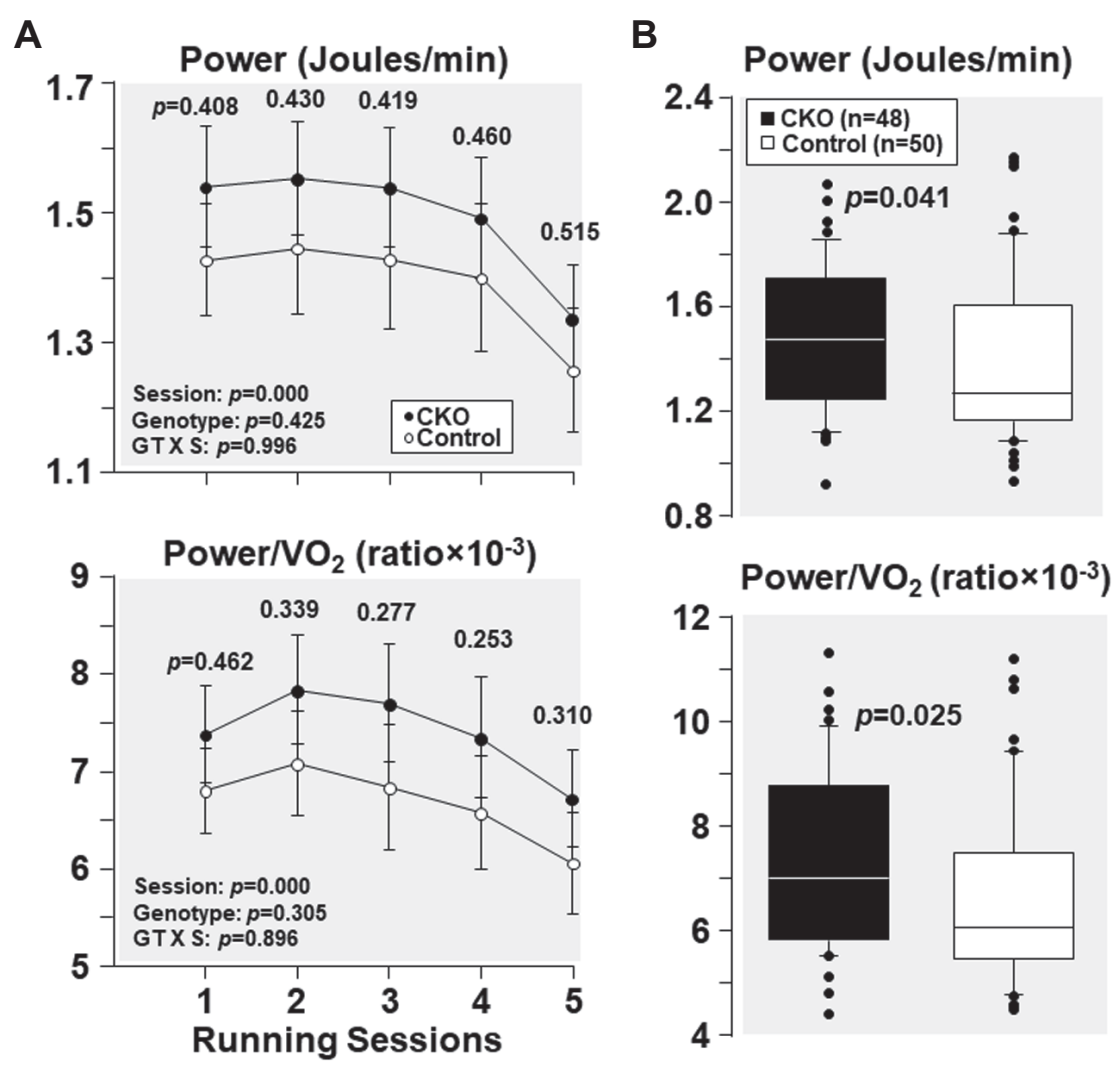
**(A)** Power and power/VO_2_, at five running sessions by conditional cyclophilin-D knockout (CKO; *n* = 10) and control mice (*n* = 10). Differences were analyzed by a linear mixed-effects model. Data from one CKO mouse were lost at running session four and five due to refusal of running. **(B)** Box plots depicting power (Joules/min) and power/VO_2_ ratio during exercise in conditional cyclophilin-D knockout (CKO) and control mice. Differences were analyzed by Mann-Whitney rank sum test.

From the first and the subsequent sessions, CKO mice had a higher power and power/VO_2_ but did not attain statistical significance (Figure 3). Yet, when all individual power and power/VO_2_ ratio throughout the five running sessions were combined, CKO mice had a statistically significant higher power and power/VO_2_ (Figure 3), consistent with increased oxygen utilization efficiency.

### Effect of Conditional Cyp-D Knockout on Fuel Utilization

**Figure 4:**
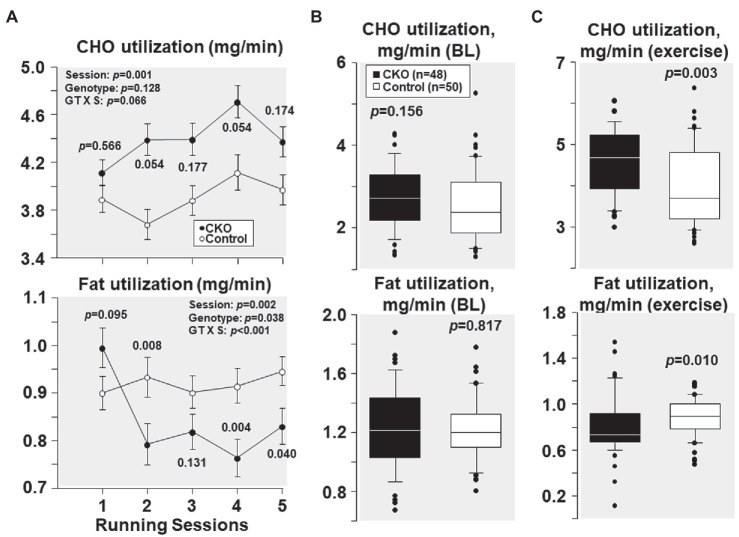
**(A)** Carbohydrate (CHO) and fat utilization at five running sessions by conditional cyclophilin-D knockout (CKO; *n* = 10) and control mice (*n* = 10). Differences were analyzed by a linear mixed-effects model. Data from one CKO mouse were lost at running session four and five due to refusal of running. **(B)** Box plots depicting estimation of carbohydrate and fat utilization by conditional cyclophilin-D knockout (CKO) and control mice at baseline (BL) and **(C)** during exercise (all individual data points of all five running sessions). Differences were analyzed by t-test when normality test passed (CHO utilization) and by Mann-Whitney Rank Sum test when normality test failed (Fat utilization).

As shown in Figure 4A, at their first running session, the fuel utilization was comparable between CKO mice and controls. Yet, with subsequent running sessions, carbohydrate utilization was higher in CKO mice but the difference was not statistically significant. Likewise, fat utilization was also comparable between the genotypes at the first running session. However, with subsequent running sessions, fat utilization was lower in CKO mice attaining statistical significance in most of the running sessions (Figure 4A).

All individual carbohydrate and fat utilization at baseline – before starting treadmill exercise – combining the five sessions demonstrated comparable carbohydrate and fat utilization in CKO mice and controls (Figure 4B). All individual carbohydrate and fat utilization during exercise throughout the five running sessions showed increased carbohydrate and decreased fat utilization in CKO mice (Figure 4C).

### Effect of Conditional Cyp-D Knockout on AMPK-TBC1D1 Nexus and Its Downstream Signaling

**Figure 5:**
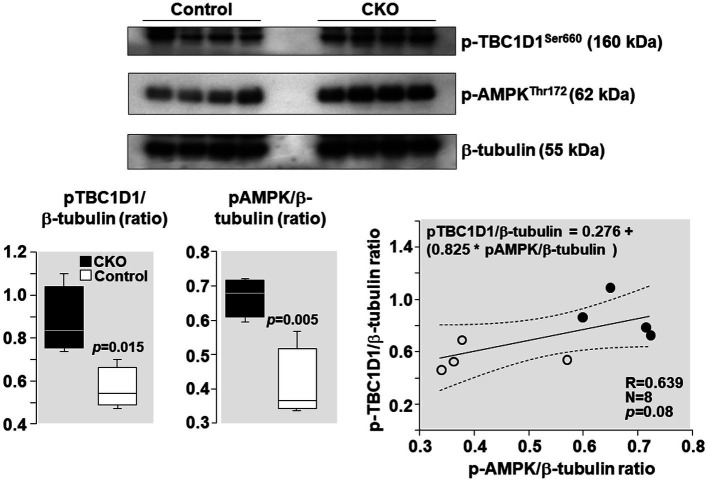
Western blots demonstrating levels of AMPK phosphorylation at threonine 172 (Thr172), TBC1D1 phosphorylation at serine 660 (Ser 660), and β-tubulin (loading control) in mice skeletal muscles after exercise. Box plots denote densitometry of pAMPK and pTBC1D1 normalized to β-tubulin. *N* = 4 from each group. Relationship between pAMPK/β-tubulin ratio and pTBC1D1/β-tubulin ratio is shown. The regression line is shown with the corresponding 95% confidence interval.

AMPK phosphorylation at Thr 172 – indicative of its activation – was increased by 64% and TBC1D1 phosphorylation Ser 660 – indicative of its inactivation – was increased by 55% in CKO mice skeletal muscles compared to control (Figure 5). Analysis of relationship between pAMPK and pTBC1D1 (Figure 5) showed a positive correlation which did not attain statistical significance (R = 0.639; *p* = 0.08).

**Figure 6:**
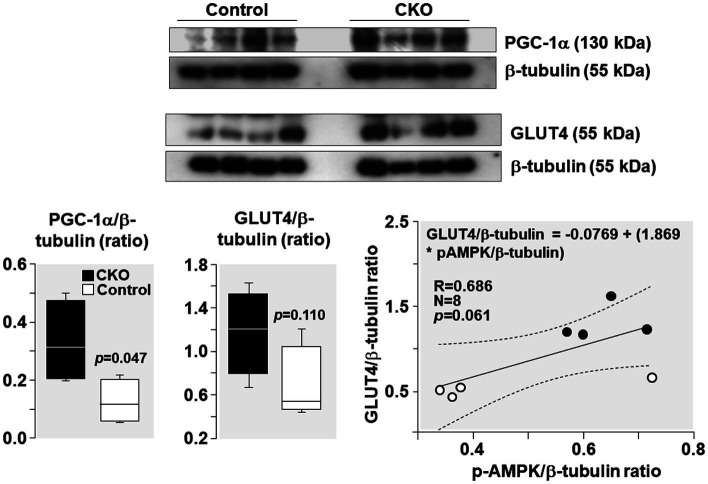
Western blots demonstrating levels of PGC-1α, GLUT4, and β-tubulin (loading control) in mice skeletal muscle after exercise. Box plots denote densitometry of PGC-1α and GLUT4 normalized to β-tubulin. *N* = 4 from each group. Relationship between pAMPK/β-tubulin ratio and GLUT4/β-tubulin ratio is shown. The regression line is shown with the corresponding 95% confidence interval.

PGC-1α levels increased by 162% and GLUT4 levels increased by 103% in CKO mice skeletal muscles compared to control (Figure 6). Analysis of relationship between GLUT4 and AMPK demonstrated a positive correlation without statistical significance (R = 0.686; *p* = 0.061).

**Figure 7:**
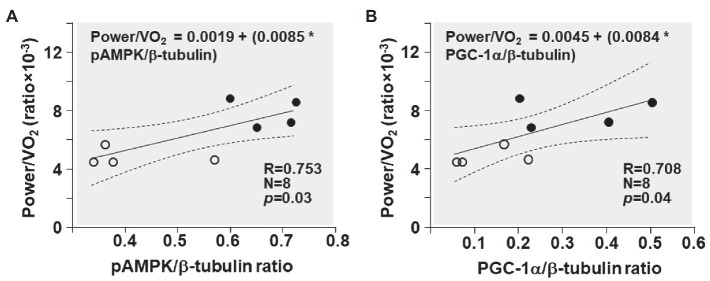
Shown are the relationships between **(A)** pAMPK/β-tubulin ratio and power/VO_2_ ratio and **(B)** PGC-1α/β-tubulin ratio and power/VO_2_ ratio in CKO mice (black circles) and controls (white circles). The regression line is shown with the corresponding 95% confidence interval.

The power/VO_2_ exhibited a positive correlation with the pAMPK/β-tubulin ratio (R = 0.753; *p* = 0.03) and the PGC-1α/β-tubulin ratio (R = 0.708; *p* = 0.04; Figure 7).

## Discussion

Consistent with our previous studies showing that Cyp-D ablation resulted in downregulation of VO_2_ in HEK 293 T cells and in constitutive KO mice, with increased oxygen utilization efficiency in the latter, the present studies demonstrate for the first time that conditional Cyp-D ablation in adult mice also reduces systemic VO_2_ and increases oxygen utilization efficiency. Reduction in VO_2_ was accompanied by higher power during treadmill exercise for a given VO_2_ and was accompanied by increased RER consistent with preferential utilization of glucose over fat. We also found direct evidence of AMPK activation with concomitant TBC1D1 inactivation and increased GLUT4 expression in skeletal muscle tissues of Cyp-D CKO mice after exercise. Taken together, these studies demonstrate that conditional Cyp-D ablation in adults leads to an adaptive response that improves oxygen utilization efficiency and exercise capacity associated with activation of AMPK signaling, an effect that invites work on options for pharmacologically recapitulating the effect, which would be beneficial under oxygen limiting conditions.

### Conditional Cyp-D Ablation Reduces VO_2_

We have previously reported in HEK 293 T cells that Cyp-D silencing results in downregulation of mitochondrial transcripts and concomitant reduction in cellular oxygen consumption (Radhakrishnan et al., 2015). Further studies in constitutive Cyp-D KO mice demonstrated downregulation of VO_2_ at rest and during endurance exercise (Radhakrishnan et al., 2019). In the present studies, we demonstrated that VO_2_ in conditional Cyp-D KO adult mice at rest and during exercise was comparable to control mice in the first running session and decreased only with subsequent sessions. Decrease in VO_2_ was accompanied by increased RER indicative of an adaptational response and training effect in CKO mice.

### Conditional Cyp-D Ablation Improves Exercise Capacity and Induces Metabolic Switch With Concomitant Activation of AMPK-TBC1D1 Signaling Nexus

During exercise, in contracting muscles, activation of AMPK occurs consequent to AMP accumulation as ATP consumption increases (Winder and Hardie, 1996). AMPK activation occurs mainly through activation of the upstream liver kinase B1(kinase LKB1) through Thr172 phosphorylation of its catalytic domain in response to increased cellular AMP:ATP ratio (Hawley et al., 2003; Woods et al., 2003). Activated AMPK inactivates its downstream target TBC1D1 (Kramer et al., 2006; Treebak et al., 2006; Taylor et al., 2008; Ferrari et al., 2019; de Wendt et al., 2021) by phosphorylation (an interaction also known as “AMPK-TBC1D1 signaling nexus”; Chavez et al., 2008; Taylor et al., 2008; Vichaiwong et al., 2010; Treebak et al., 2014; Kjøbsted et al., 2016; Ferrari et al., 2019). TBC1D1 inactivation during exercise has been reported in mouse skeletal muscles (Taylor et al., 2008; Vichaiwong et al., 2010; de Wendt et al., 2021) and in human skeletal muscles (Jessen et al., 2011; Tobias et al., 2020). Activation of AMPK-TBC1D1 signaling nexus results in increased GLUT4 expression and translocation from the cytosol to the plasma membrane enabling increased glucose uptake. Glucose enters the glycolytic pathway and is metabolized in the cytosol to pyruvate and then to lactate by lactate dehydrogenase (LDH). The conversion of pyruvate to lactate is favored over alternative pyruvate fates given the near-equilibrium nature of the LDH reaction (Lambeth and Kushmerick, 2002) and a much higher activity than regulatory enzymes of the glycolytic and oxidative pathways (Rogatzki et al., 2015). Lactate then enters the mitochondria where it is converted to pyruvate by the LDH present on the mitochondrial inner membrane (Hashimoto et al., 2006; Gladden, 2008). Pyruvate is then converted to acetyl coA and metabolized in the Krebs cycle. Thus, the AMPK-TBC1D1 signaling nexus increases glucose uptake and enhances glucose utilization through glycolysis and the Krebs cycle. Consistent with this effect, we observed in the present study activation of AMPK-TBC1D1 signaling nexus (Figure 5) and increased GLUT4 expression (Figure 6) in CKO mice after exercise. Consistently, several other studies have also showed increased expression of skeletal muscle GLUT4 in rats and human after exercise (Ivy et al., 1999; Kuo et al., 1999; MacLean et al., 2000; Flores-Opazo et al., 2020). Increased GLUT4 expression has been correlated with increased glucose transport and utilization (Kern et al., 1990; Ivy et al., 1999; MacLean et al., 2000).

In addition, AMPK activation also increases the expression of PGC-1α (as also shown in Figure 6). The effect occurs by phosphorylation at its threonine-177 and serine-538 residues prompting self-dependent activation of its promoter and gene expression (Jäger et al., 2007). PGC-1α further increases GLUT4 expression and thereby glucose uptake (Jäger et al., 2007). In addition, PGC-1α activates mitochondrial biogenesis and other pathways, which could improve the exercise capacity after training. Several lines of evidence suggest that PGC-1α is a key factor in mediating exercise training induced adaptations in mitochondria (Olesen et al., 2010) in response to AMPK activation. For example, AICAR – the AMPK activator – improves exercise capacity in mice with concomitant increase in PGC-1α gene expression (Narkar et al., 2008). In addition, AICAR injections fail to increase levels of mitochondrial and metabolic proteins in skeletal muscles of PGC-1α KO mice (Leick et al., 2010). Thus, AMPK in CKO mice could induce additional adaptive responses, including mitochondrial biogenesis *via* PGC-1α, leading to enhanced exercise capacity.

In our previous studies in constitutive Cyp-D KO mice, the aforementioned metabolic effects were associated with downregulation of electron transport complexes I and IV activities consequent to reduced expression of subunits encoded by the mitochondrial heavy strand promoter 2 (Radhakrishnan et al., 2015, 2019). The expression of these subunits requires the interaction of Cyp-D with the mitochondrial transcription factors B1 and B2 (Radhakrishnan et al., 2015). The reduced activity of electron transport complexes I and IV would create an “energy-stress” (Gowans et al., 2013) during exercise prompting an increase in AMP:ATP ratio and the consequent activation of the AMPK-TBC1D1 signaling nexus. Other studies have also reported complex I inhibition as the mechanism of AMPK activation. In fact, the mechanism of pharmacological activation of AMPK by metformin involves electron complex I inhibition (Owen et al., 2000; Zhou et al., 2001; Mohsin et al., 2019). However, metformin abrogates exercise-mediated increase in skeletal muscle mitochondrial respiration (Konopka et al., 2019).

In our previous study in constitutive Cyp-D KO mice, we have documented a metabolic shift – i.e., increased glucose utilization over fat. Tavecchio et al. (2015) have documented in their study that Cyp-D constitutive KO mice had increased transcription of genes involved in glucose metabolism resulting in increased glucose utilization and impaired fatty acid utilization. In the present study in conditional Cyp-D KO mice, we have documented a similar adaptive response. In the present study, AMPK activation was accompanied by increased glucose utilization over fatty acids (as shown in Figure 4) that is energetically advantageous. Glucose oxidation per mole yields 6.3 moles of ATP, whereas fatty acid yields only 5.6 moles of ATP (Opie, 1991; Stanley and Chandler, 2002) resulting in a 12.5% increase in energy production with glucose oxidation. Thus, this metabolic switch enhances oxygen utilization efficiency during exercise and improves exercise capacity as evidenced by the significant increase in power during treadmill exercise for given oxygen consumed (power/VO_2_ ratio; as shown in Figure 3).

### Comparison Between Constitutive and Conditional Cyp-D KO

Both constitutive and conditional Cyp-D KO had their Cyp-D knocked-out in all tissues. Cyp-D ablation was carried out at an embryonic stage in constitutive KO, whereas Cyp-D ablation was carried out at the adult stage in CKO mice. Yet, the CKO mice displayed similar effects – VO_2_ downregulation and metabolic switch – as that of constitutive KO but of lesser magnitude. The metabolic switch in CKO mice was prominent at the second running session and thereafter, as documented by increased glucose utilization over fatty acids (Figure 4). The first and second running sessions were performed at the third and the fourth week, respectively, after tamoxifen treatment for 5 days. This hints to the possibility that the metabolic switch can be triggered probably only 4 weeks after completion of pharmacological interventions. The time delay for this metabolic switch – we rationalize – includes the time required for cre/lox-P recombination-dependent Cyp-D ablation and subsequent respiratory chain downregulation. In addition, exercise training induced mitochondrial adaptations involving PGC-1α could also have played role in improving the exercise capacity. These results point to the fact that selectively destabilizing interaction between Cyp-D and mitochondrial transcription factors to reduce oxygen consumption without affecting other functions of Cyp-D in adults could have potential positive impact on oxygen utilization efficiency and it could be highly beneficial under oxygen limiting conditions.

### Conclusion

Conditional Cyp-D ablation reduced VO_2_ and increased power/VO_2_ ratio, demonstrating increased oxygen utilization efficiency consistent with our previous work in constitutive KO mice. Conditional Cyp-D ablation also triggered the adaptive response which involved a metabolic switch toward increased glucose utilization with concomitant activation of AMPK-TBC1D1 signaling nexus and increased GLUT4 expression in skeletal muscles after exercise. Our study raises the possibility that the metabolic switch can probably be triggered in 4 weeks after completion of pharmacological intervention. Developing tools for modulation of this adaptive response could be beneficial in a myriad of physiological and clinical conditions where oxygen availability is limited.

## Data Availability Statement

The raw data supporting the conclusions of this article will be made available by the authors, without undue reservation.

## Ethics Statement

The animal study was reviewed and approved by the Institutional Animal Care and Use Committee at Rosalind Franklin University of Medicine and Science (Protocol B17-20) and by the Edward Hines VA Hospital Institutional Animal Care and Use Committee (Protocol H17-014).

## Author Contributions

JR and RJG conceived and designed the research and wrote the manuscript and agreed on the final version. JR performed the experiments with the assistance of AB. AB developed the software necessary for the experiments and data analysis. JR and AB analyzed the data. All authors contributed to the article and approved the submitted version.

## Funding

We are thankful for the funding support by the VA Merit Review Award (1 I01 BX002771-01) to RJG.

## Conflict of Interest

The authors declare that the research was conducted in the absence of any commercial or financial relationships that could be construed as a potential conflict of interest.

## Publisher’s Note

All claims expressed in this article are solely those of the authors and do not necessarily represent those of their affiliated organizations, or those of the publisher, the editors and the reviewers. Any product that may be evaluated in this article, or claim that may be made by its manufacturer, is not guaranteed or endorsed by the publisher.
